# Insecticidal activity and the mechanism of action of three phenylpropanoids isolated from the roots of *Piper sarmentosum* Roxb

**DOI:** 10.1038/s41598-017-12898-z

**Published:** 2017-10-03

**Authors:** Arshia Hematpoor, Sook Yee Liew, Mohd Sofian Azirun, Khalijah Awang

**Affiliations:** 1 Institute of Biological Sciences, Faculty of Science, University of Malaya, 50603 Kuala Lumpur, Malaysia; 2 Department of Chemistry, Faculty of Science, University of Malaya, 50603 Kuala Lumpur, Malaysia; 3 Centre for Natural Products and Drug Discovery (CENAR), University of Malaya, 50603 Kuala Lumpur, Malaysia

## Abstract

Hexane, dichloromethane and methanol extracts of the roots of *Piper sarmentosum* Roxb. were screened for toxicity towards *Sitophilus oryzae* (L.), *Rhyzopertha dominica* (F.), and *Plodia interpunctella* (Hübner) and the hexane extract exhibited the highest mortality percentage. Bioassay-guided fractionation of the hexane extract resulted in the isolation of asaricin **1**, isoasarone **2**, and *trans*-asarone **3**. Asaricin **1** and isoasarone **2** were the most toxic compounds to *Sitophilus oryzae*, *Rhyzopertha dominica*, and *Plodia interpunctella*. *Sitophilus oryzae* and *Rhyzopertha dominica* exposed to asaricin **1** and isoasarone **2** required the lowest median lethal time. Insecticidal activity of *trans*-asarone **3** showed consistent toxicity throughout the 60 days towards all three insects as compared to asaricin **1** and isoasarone **2**. Asaricin **1** and isoasarone **2** at different doses significantly reduced oviposition and adult emergence of the three insects in treated rice. *Trans*-asarone **3** had lowest toxicity with highest LC and LT values in all tested insects relative to its mild oviposition inhibition and progeny activity. Moreover, asaricin **1** and isoasarone **2** significantly inhibited acetylcholinesterase in comparison with *trans*-asarone **3** and the control. Acetylcholinesterase inhibition of *Rhyzopertha dominica* and *Plodia interpunctella* by asaricin **1** and isoasarone **2** were lower than that of *Sitophilus oryzae*, which correlated with their higher resistance.

## Introduction

Storage pests have a direct effect on the reduction of the quantity and quality of grain during postharvest storage. The damages brought upon stored grains and their related grain products by insects could be as high as 20–30% in tropical countries such as Malaysia^1–3^. *Sitophilus oryzae* (L.), *Rhyzopertha dominica* (F.), and *Plodia interpunctella* (Hübner) are considered as main pests of stored food^4^. These insects can infest a wide range of stored grains such as wheat and corn. The adults of *S. oryzae* and *R. dominica* are known to attack and consume the intact grains while their larvae feed on the kernel and develop inside of it^5,6^. *Plodia interpunctella*, also known as the Indian moth larva, during its adult stage does not cause much damage as compared to its larval stage^7^. *P. interpunctella* is an external feeder. The larva continuously produces a silk web around the surface and inside of the food and feeds within the web^8^.

Synthetic insecticides are commonly used to protect stored grains and their related products against pests. The mechanism of toxicity of most insecticides such as organophosphorus and carbamate compounds is based on the inhibition of acetylcholinesterase^9–11^. In insects, acetylcholinesterase (AChE) hydrolyzes the neurotransmitter acetylcholine (ACh) to terminate neuronal excitement at the postsynaptic membrane^12^. Repeated usage of synthetic insecticides has resulted in poisoning non-targeted organisms, residual contamination and the increased resistance in insect pests^13–15^. These problems have warranted the need for developing alternative strategies which include using ecofriendly products in particular plant derived compounds^16–18^.

Plants offer an alternative source of insect-control agents as they contain a wide range of bioactive compounds, many of which are selective and have little or no harmful effects on non-targeted organisms and the environment unlike synthetic insecticides^16,19,20^. Plant extracts have been employed in many traditions as insecticides even before the advent of synthetic insecticides. Their potential in controlling insect growth has been used to store grains^21,22^. For example, farmers in India used neem leaves and seed to control stored grain pests^23,24^ and in some traditions in East Africa, dried leaves of *Ocimum* plants are added to foodstuff to protect against pests^25^.

*Piper sarmentosum* Roxb. is mainly distributed in tropical and sub-tropical regions of the world. It has an aromatic odour and a pungent taste^26,27^. In Malaysia, it is known as ‘kadok’^28^ and often used for traditional treatment of a variety of ailments such as cough, headache, fungal dermatitis and pleurisy^27^. Since the genus *Piper* is an important source of secondary metabolites which exhibit insecticidal activity^29^, hence, the metabolites have potential as pest control agents^30^. For example, the extracts and chemical constituents of *P*. *sarmentosum* are known to demonstrate larvicidal activity against mosquitoes^31–33^. However, to the knowledge of the author, there is no report on its potential insecticidal activity against storage pests. Hence, this prompted us to investigate the insecticidal activity of the hexane, dichloromethane and methanol extracts of *P*. *sarmentosum* against three important stored rice pests, namely *S. oryzae*, *R. dominica*, and *P. interpunctella*. Subsequently, a bioassay-guided fractionation of the active extract was conducted in order to isolate and characterize the compounds which were responsible for its toxicity towards *S. oryzae*, *R. dominica*, and *P. interpunctella*. Activity levels of the isolated compounds on the inhibition of AChE and its target site were monitored using biochemical assays.

## Results

### Insecticidal activity with treated grains

**Figure 1:**
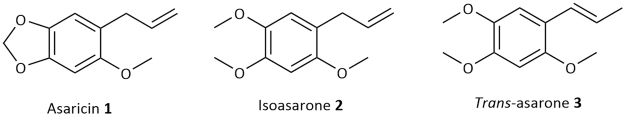
Isolated phenylpropanoids; asaricin **1**, isoasarone **2**, and *trans*-asarone **3**.

The toxicity potential of the hexane (HE), dichloromethane (DE) and methanol extracts (ME) from the roots of *P. sarmentosum* against *S. oryzae*, *R. dominica*, and *P. interpunctella* are presented in Table 1. Based on the results obtained, the hexane extract exhibited the highest percentage of mortality for all three storage pests at a concentration level of 500 µg/mL over an exposure period of 72 hours (Table 1). Hence, HE was subjected to toxicity-guided fractionation which gave faction 2 (F2) as the active fraction (Table 1). Potent toxicity of F2 led to the isolation and characterization of asaricin **1**, isoasarone **2**, and *trans*-asarone **3** as the active constituents (Fig. 1).

**Table 1:** Percentage of mortality of *S. Oryzae*, *R. dominica *adults and *P. interpunctella* larvae 72 hours after exposure to 500 µg/mL of extracts and fractions.

Tested extracts and fractions*	% Mortality (mean ± SE) after 72 hours**		
	*S. oryzae*	*R. dominica*	*P. interpunctella*
HE	100.0 ± 0.0 a	87.8 ± 3.9 b	59.1 ± 4.6 b
DE	9.6 ± 3.9 c	7.1 ± 1.7 d	4.3 ± 2.4 d
ME	76.8 ± 3.9 b	57.4 ± 6.1 c	32.1 ± 4.4 c
F2	100.0 ± 0.0 a	100.0 ± 0.0 a	82.8 ± 6.8 a

*HE (Hexane extract), DE (dichloromethane extract), ME (Methanol extract), F2 (Fraction two out of eight fractions of the hexane extract).

**Mean ( ± SE) followed by the same letters in a row indicate no significant difference (*p < *0.05) according to the Tukey test.

Fractions with no activity were not presented.

### Evaluation of lethal concentration of the compounds

Asaricin **1** and isoasarone **2** showed high toxicity against all three tested insects. The LC_50_ of *S. oryzae* was estimated to be 4.7 µg/mL for asaricin **1** and 5.6 µg/mL for isoasarone **2** which considered the lowest LC_50_ among the tested insects (Table 2). Similarly lowest LC_95_ value was observed for *S. oryzae* in response to asaricin **1** (13.6 µg/mL) followed by isoasarone **2** (14.3 µg/mL)*. P. interpunctella* was more tolerant to the toxicity of asaricin **1** and isoasarone **2** with LC_95_ value of 35.9 and 37.7 µg/mL respectively.

**Table 2:** LC_50_ and LC_95_ of asaricin **1**, isoasarone **2**, and *trans*-asarone **3** against *S. oryzae, R. dominica* and *P. interpunctella* after 72 hours exposure.

Compounds*	Tested Insects**											
	*S. oryzae*				*R. dominica*				*P. interpunctella*			
	LC_50_ (µg/mL) (95% C.I.)***	LC_95_ (µg/mL) (95% C.I.)***	F****	*p*	LC_50_ (µg/mL) (95% C.I.)***	LC_95_ (µg/mL) (95% C.I.)***	F****	*p*	LC_50_ (µg/mL) (95% C.I.)***	LC_95_ (µg/mL) (95% C.I.)***	F****	*p*
Asaricin **1**	4.70 (3.6 to 5.6)	13.60 (10.5 to 21.6)	15.2	<0.05	10.60 (8.1 to 11.9)	18.60 (15.8 to 30.3)	8.4	<0.05	17.37 (13.4 to 20.3)	35.90 (29.1 to 57.1)	15.3	<0.05
Isoasarone **2**	5.60 (4.5 to 6.6)	14.30 (11.5 to 20.8)	11.3	<0.05	8.70 (7.16 to 10.6)	28.10 (20.3 to 49.2)	10.8	<0.05	15.76 (12.4 to 19.1)	37.70 (29.1 to 61.5)	11.5	<0.05
*Trans*-asarone **3**	258.90 (218.7 to 303.1)	670.20 (531.1 to 967.7)	25.0	<0.05	396.48 (328.1 to 474.7)	1283.56 (959.6 to 2072.3)	32.1	<0.05	432.42 (387.3 to 485.2)	1795.70 (1425.1 to 2444.6)	4.05	<0.05

*Asaricin **1**, isoasarone **2** and *trans*-asarone **3** represent phenylpropanoids isolated from *P. sarmentosum* hexane active fraction (F2).

**Test performed on *S. oryzae* and *R. dominica* two weeks old adults and *P. interpunctella* third instar larvae.

***LC_50_ and LC_95_ values significant difference (*p* < 0.05) is based on non-overlap of the 95% confidence interval (C.I.).

****Since Chi square goodness of fit test is not significant (*p* > 0.15), no heterogeneity factor is used in the calculation of fiducial limits.

### Evaluation of lethal time of the compounds

The lethal time (LT_50_ and LT_95_) by each isolated compound studied on *S. oryzae*, *R. dominica*, and *P. interpunctella* was presented in Table 3. Smaller LT_50_ and LT_95_ values of asaricin **1** and isoasarone **2** indicated both compounds had faster action than *trans*-asarone **3**. However, *S. oryzae* and *R. dominica* exposed to asaricin **1** and isoasarone **2** had lower LT_50_ values ranging from 15.7 hours to 18.4 hours as compared to *P. interpunctella* (46.9 hours). *Trans*-asarone **3** showed the highest LT_50_ (≥38.9 hours) and LT_95_ values (≥79.2 hours). By comparing of 95% confidence interval there was no significant difference (*p* > 0.05) between the LT values observed from asaricin **1** and isoasarone **2**. *P. interpunctella* had the significant highest LT values as compared to *S. oryzae* and *R. dominica*.

**Table 3:** LT_50_ and LT_95_ of asaricin **1**, isoasarone **2**, and *trans*-asarone **3** against *S. oryzae, R. dominica* and *P. interpunctella* after 72 hours exposure.

Compounds	Tested Insects*											
	*S. oryzae*				*R. dominica*				*P. interpunctella*			
	LT_50_ (hours) (95% C.I.)**	LT_95_ (hours) (95% C.I.)**	F***	*p*	LT_50_ (hours)(95% C.I.)**	LT_95_ (hours)(95% C.I.)**	F***	*p*	LT_50_ (hours) (95% C.I.)**	LT_95_ (hours) (95% C.I.)**	F***	*p*
Asaricin **1**	15.7 (8.6 to 19.6)	34.2 (22.5 to 51.2)	7.8	<0.05	18.4 (11.1 to 27.8)	39.8 (29.8 to 57.7)	12.1	<0.05	46.9 (28.7 to 62.2)	91.5 (63.1 to 167.3)	9.9	<0.05
Isoasarone **2**	17.3 (9.5 to 23.4)	39.3 (27.3 to 56.8)	12.8	<0.05	19.6 (11.2 to 28.6)	48.3 (31.2 to 61.5)	10.5	<0.05	45.3 (34.4 to 51.8)	88.1 (69.6 to 159.6)	13.2	<0.05
*Trans*-asarone **3**	38.9 (28.8 to 43.5)	79.2 (58.2 to 103.4)	28.4	<0.05	48.7 (31.6 to 57.2)	88.9 (68.9 to 129.2)	30.3	<0.05	52.7 (39.4 to 82.9)	92.7 (88.1 to 134.9)	18.8	<0.05

*Test performed on *S. oryzae* and *R. dominica* two weeks old adults and *P. interpunctella* their instar larvae.

**LT_50_ and LT_95_ values significant difference (*p* < 0.05) is based on non-overlap of the 95% C.I.

***Since Chi square goodness of fit test is not significant (*p* > 0.15), no heterogeneity factor is used in the calculation of fiducial limits.

### Contact toxicity

**Figure 2:**
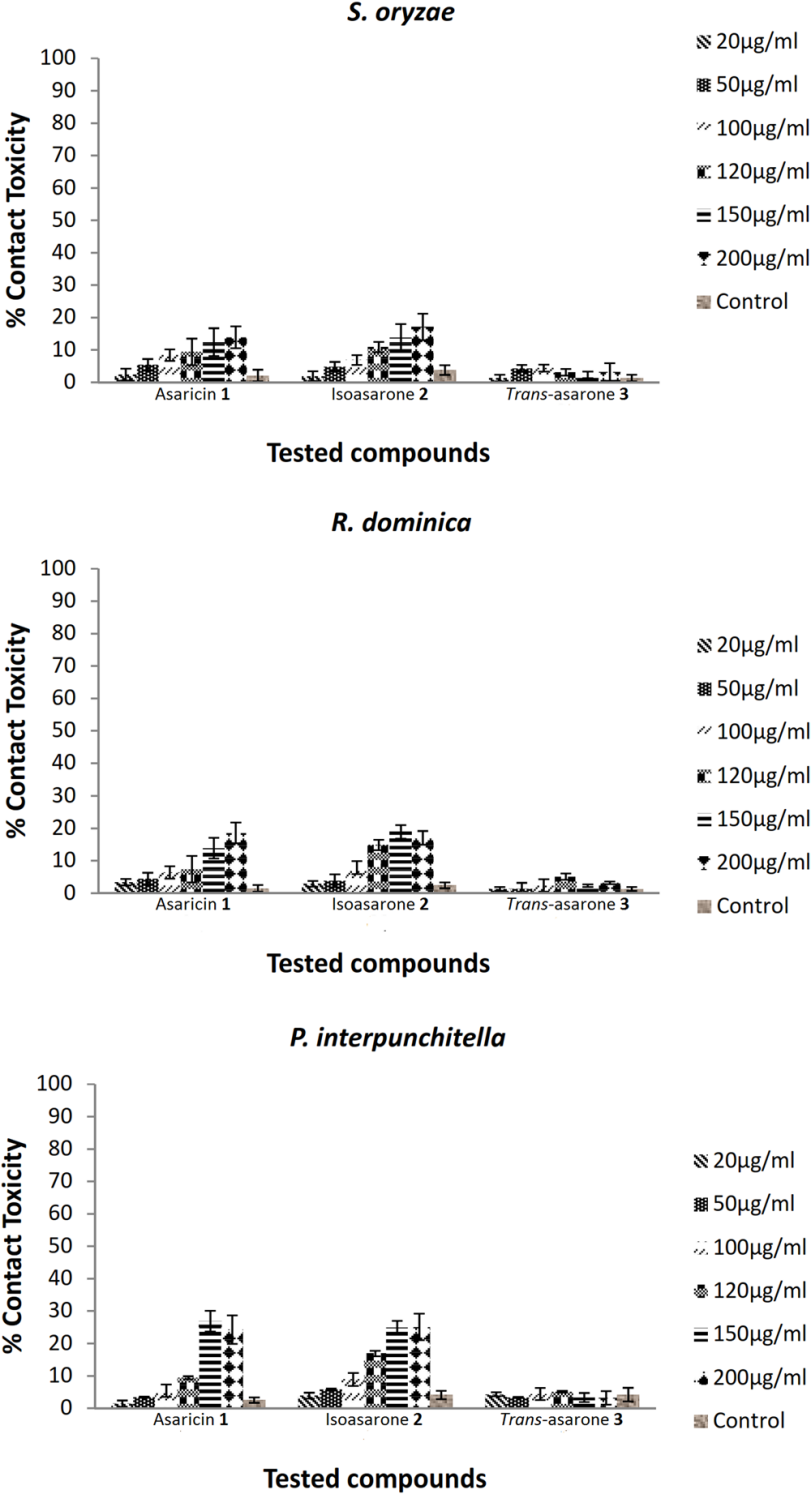
Percentage of contact toxicity of asaricin **1**, isoasarone **2**, and trans-asarone **3** against *S. oryzae* and *R. dominica* adults and *P. interpunctella* larvae. Data were expressed as mean ± SEM.

The results clearly demonstrated that asaricin **1**, isoasarone **2**, and *trans*-asarone **3** did not show potent contact toxicity against stored grain insect pest even at the highest concentration of 200 μg/mL (Fig. 2). The efficacy in respect to the toxicity of asaricin **1**, isoasarone **2**, and *trans*-asarone **3** towards *S. oryzae* and *R. dominica* adults and *P*. *interpunctella* larvae was relatively weak up to 48 hours monitoring after the treatment. *Trans*-asarone **3** were not significantly different as compared to the control, *S. oryzae* (*F* = 3.3, *p* < 0.05), *R. dominica* (*F* = 3.8*, p* < 0.05) and *P*. *interpunctella* (*F* = 3.8, *p* < 0.05).

### Residual toxicity using LC_95_ value of each active compound

**Figure 3:**
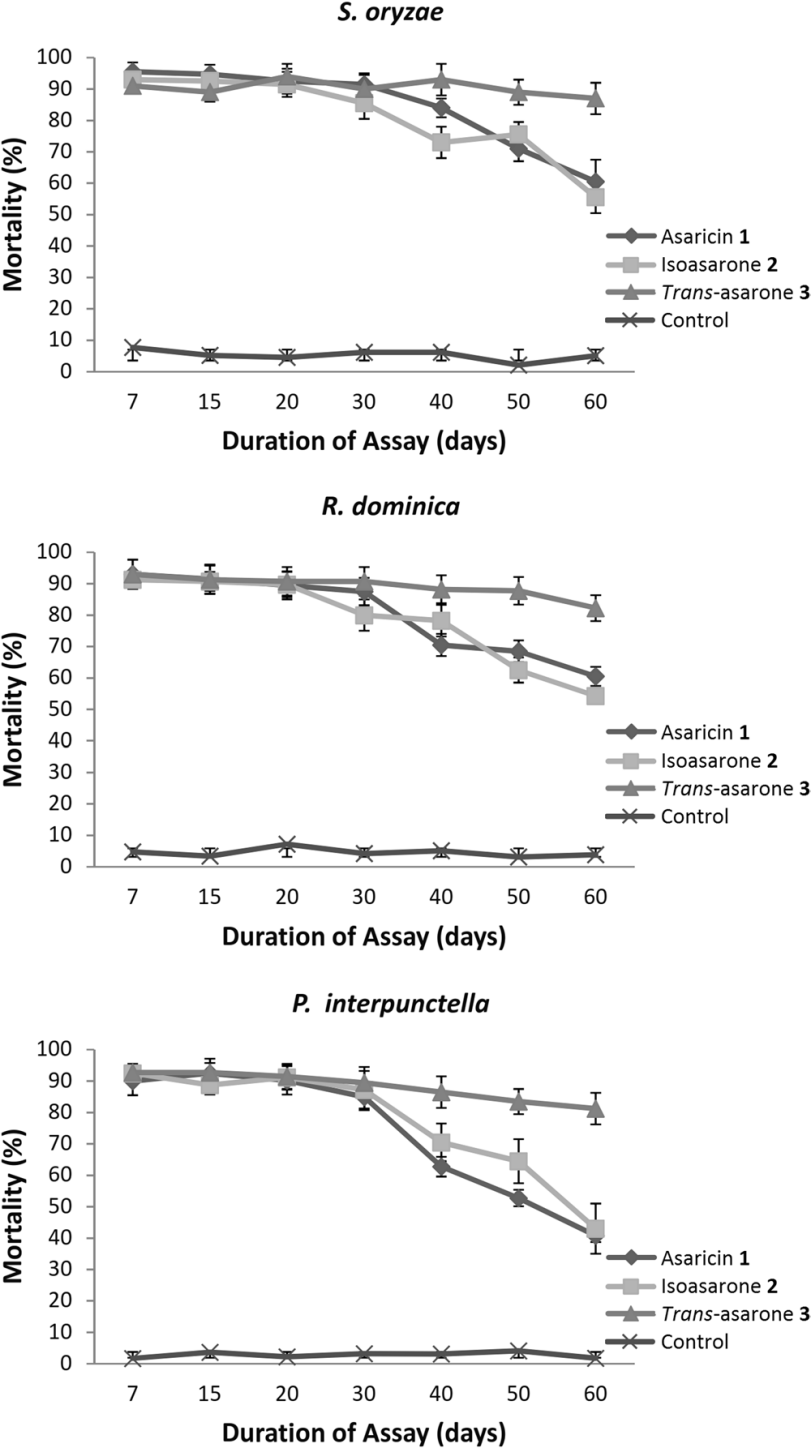
Mean (±SEM) mortality percentage of *S. oryzae, R. dominica* adult and *P. interpunctella* larvae on rice grain treated with asaricin **1**, isoasarone **2**, and *trans*-asarone **3** by using their relative LC_95_ value, exposed from 0 to 60 days after treatment. Percentage of mortality for *trans*-asarone **3** were significant stable as compared to asaricin **1** and isoasarone **2** after 30^th^ days.

The mean mortality of *S. oryzae, R. dominica* and *P. interpunctella* were recorded during the 60 days of bioassay (Fig. 3). During the 60 days of residual activity test, the mean mortality of *S. oryzae* (*F* = 3.6*, p *< 0.05), *R. dominica* (*F* = 4.6*, p* < 0.05) and *P. interpunctella* (*F* = 4.4, *p* < 0.05) were significantly higher than the control after treated with asaricin **1**, isoasarone **2** and trans-asarone **3**. The efficacy of asaricin **1** and isoasarone **2** with their relative LC_95_ against *S. oryzae* was consistent during the first 30 days and slowly decreased and this pattern continued till last day of the assay. On the other hand, toxicity efficacy of *trans*-asarone **3** towards *S. oryzae* with high LC_95_ value of 670.2 μg/mL was consistent till the 60^th^ day. *R. dominica* and *P. interpunctella* response to residual effects of asaricin **1**, isoasarone **2**, and *trans*-asarone **3** were similar and followed almost the same pattern as *S. oryzae*. Asaricin **1** and isoasarone **2** showed consistent toxicity against tested insects during the first 30 days according to the mortality rate. Then, the insecticidal activity reduced and by the 60^th^ day, it was at the lowest point (Fig. 3).

### Repellency bioassay

Asaricin **1** and isoasarone **2** showed similar repellency activity towards *S. oryzae, R. dominica*, and *P. interpunctella* (Table 4)*. S. oryzae* and *R. dominica* were repelled more by asaricin **1** and isoasarone **2** with mean repellency of 79–85% while *P. interpunctella* was less repelled with mean repellency of 50–58% after 20 hours. *Trans*-asarone **3** was less effective in repellency with less than 10% repellency for all tested insects.

**Table 4:** Repellency activity of asaricin **1**, isoasarone **2**, and *trans*-asarone **3** against *S. oryzae, R. dominica* and and *P.interpunctella*.

Compounds	Insects	Repellency (mean ± SE)*				Mean repellency (%)
		5 h	10 h	15 h	20 h	
Asaricin **1**	*S. oryzae*	51.2 ± 10.3 a	67.2 ± 8.5 a	74.2 ± 5.3 ab	82.5 ± 6.4 a	68.6 ± 6.6 a
	*R. dominica*	60.0 ± 7.1 a	74.5 ± 6.4 a	81.2 ± 6.3 a	85.0 ± 7.8 a	75.1 ± 5.5 a
	*P. interpunctella*	33.7 ± 4.7 b	38.7 ± 7.5 b	45.4 ± 7.2 c	50.0 ± 7.1 b	42.7 ± 3.9 b
Isoasarone **2**	*S. oryzae*	51.2 ± 7.5 a	60.0 ± 7.8 a	69.5 ± 6.2 b	79.2 ± 8.3 a	68.1 ± 6.2 a
	*R. dominica*	58.7 ± 13.1 a	65.0 ± 7.9 a	72.5 ± 5.5 ab	79.2 ± 5.1 a	68.8 ± 4.4 a
	*P. interpunctella*	31.2 ± 2.5 b	37.5 ± 6.5 b	41.2 ± 2.5 c	58.7 ± 4.8 b	42.1 ± 5.8 b
*Trans*-asarone **3**	*S. oryzae*	NA	NA	NA	NA	NA
	*R. dominica*	NA	NA	NA	NA	NA
	P. interpunctella	**NA**	**NA**	**NA**	**NA**	**NA**

*Each data represents the mean of four replicates. Values followed by the same letter are not significantly different according to the Tukey test (*p* < 0.05).

h: hours after treatment.

NA: below 10% of repellency.

### F1 progeny activity

Table 5 shows the effect of F1 progeny activity of asaricin **1**, isoasarone **2**, and *trans*-asarone **3** on *S. oryzae*, *R. dominica*, and *P. interpunctella*. Asaricin **1** and isoasarone **2** were highly potent on inhibition of *S. oryzae, R. dominica*, and *P. interpunctella* emergence of the adult insects at low dosage as 2 µg/mL. The asaricin **1** and isoasarone **2** at 4 µg/mL concentrations were able to inhibit 100% of the emergence of adult *S. oryzae* and *R. dominica*. *P. interpunctella* were more resistance to asaricin **1** and isoasarone **2** where they only caused 78.98 and 86.37% inhibition of F1 adult emergence at 4 µg/mL. Meanwhile *trans*-asarone **3** was weak in inhibition of F1 adult emergence as compared to asaricin **1** and isoasarone **2**. All the tested isolated compounds were significantly (*p* < 0.05) different from the controls in their effects.

**Table 5:** Effect of asaricin **1**, isoasarone **2**, and *trans*-asarone **3** against on the adult emergence of three stored product insect pests infesting rice.

Compounds	Concentrations (µg/mL)	Percentage of reduction in egg number					
		S. oryzae*		R. dominica*		P. interpunctella*	
		Mean number of adult emergence	% IR**	Mean number of adult emergence	% IR**	Mean number of adult emergence	% IR**
Asaricin **1**	1	19.25 ± 2.01	34.25 ± 1.30 d	16.75 ± 2.17	41.10 ± 1.80 c	28.50 ± 0.86	21.50 ± 2.25 d
	2	2.08 ± 1.04	89.25 ± 3.70 b	1.52 ± 0.76	84.50 ± 2.90 b	11.50 ± 2.36	59.25 ± 3.83 c
	4	0.00 ± 0.00	100.00 ± 0.00 a	0.00 ± 0.00	100.00 ± 0.00 a	3.80 ± 1.20	87.37 ± 3.47 a
Isoasarone **2**	1	15.00 ± 2.64	47.50 ± 1.47 c	15.50 ± 2.53	44.70 ± 1.40 c	21.50 ± 1.52	24.60 ± 2.10 d
	2	1.52 ± 0.76	86.00 ± 3.20 b	1.73 ± 0.86	89.34 ± 2.80 b	11.50 ± 1.52	59.50 ± 2.30 c
	4	0.00 ± 0.00	100.00 ± 0.00 a	0.00 ± 0.00	100.00 ± 0.00 a	5.40 ± 0.98	78.98 ± 4.51 b
*Trans*-asarone **3**	1	28.00 ± 1.54	2.25 ± 0.71 f	25.30 ± 0.50	1.00 ± 0.40 f	25.50 ± 1.52	1.17 ± 1.20 g
	2	25.00 ± 1.52	13.50 ± 2.10 e	25.75 ± 2.88	17.75 ± 0.83 d	24.50 ± 2.40	7.50 ± 0.04 f
	4	26.50 ± 1.54	8.60 ± 1.02 e	28.50 ± 1.73	9.25 ± 1.04 e	26.25 ± 2.64	14.25 ± 2.50 e

*Test performed on *S. oryzae* and *R. dominica* two weeks old adults and *P. interpunctella* their instar larvae.

**% IR ﻿(inhibition rate)﻿ values followed by a common letter are not significantly different (*p* < 0.05).

### Oviposition deterrent effect

**Figure 4:**
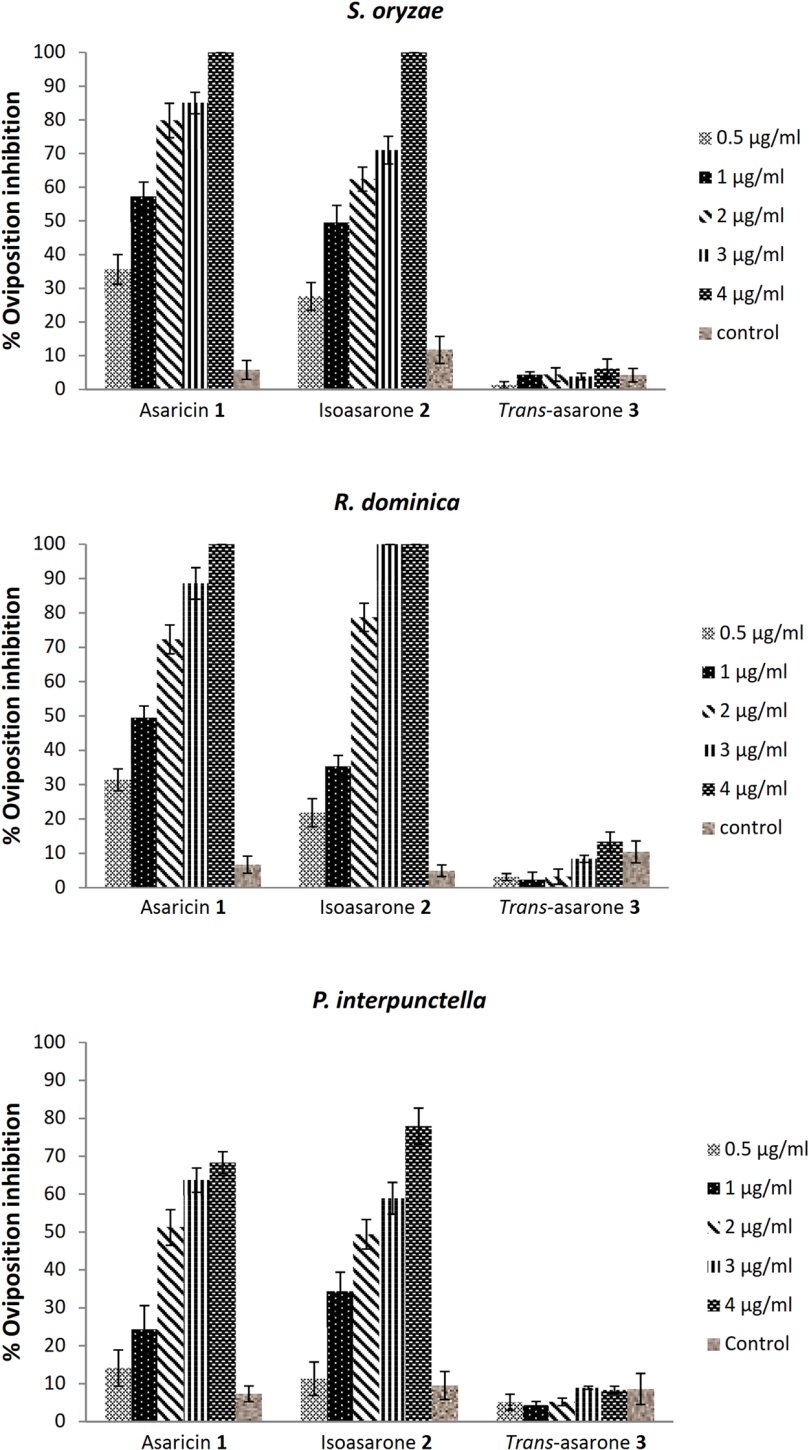
Oviposition inhibition of asaricin **1**, isoasarone **2**, and *trans*-asarone **3** on *S. oryzae, R.dominica* and *P. interpunctella*.

The ovipositional inhibition effect is demonstrated in Fig. 4. The egg deposition was recorded 72 hours after exposure of insects to asaricin **1**, isoasarone **2**, and *trans*-asarone **3**. Cumulative number of egg laying by the *S. oryzae*, *R. dominica*, and *P. interpunctella* females were highly inhibited by asaricin **1** and isoasarone **2**. These results indicated that asaricin **1** and isoasarone **2** significantly suppressed the egg deposition tested insects. Asaricin **1** and isoasarone **2** caused 10 to 35% reduction of egg deposition at the lowest dosage of 0.5 µg/mL in all tested insects. At concentration of 4 µg/mL, asaricin **1** and isoasarone **2** caused 100% ovipositional inhibition in *S. oryzae* and *R. dominica* and up to 68–77.9% inhibition in *P. interpunctella*. Overall, the statistical analysis of proportions of egg laying by insect pests between control and asaricin **1** were found to be statistically significant for *S. oryzae* (*F* = 7.6*, p* > 0.05), *R. dominica* (*F* = 5.1, *p* > 0.05) and *P. interpunctella* (*F* = 4.4, *p* > 0.05) which was similar to isoasarone **2**. On the other hand, *trans*-asarone **3** had weak ovipositional inhibition in all tested insects where it could not reduce the egg laying after treatment. There was no significant differences in *trans*-asarone **3** and control (*F* = 3.9, *p* < 0.05).

### Cholinesterase Enzymes Inhibitory Activity

**Figure 5:**
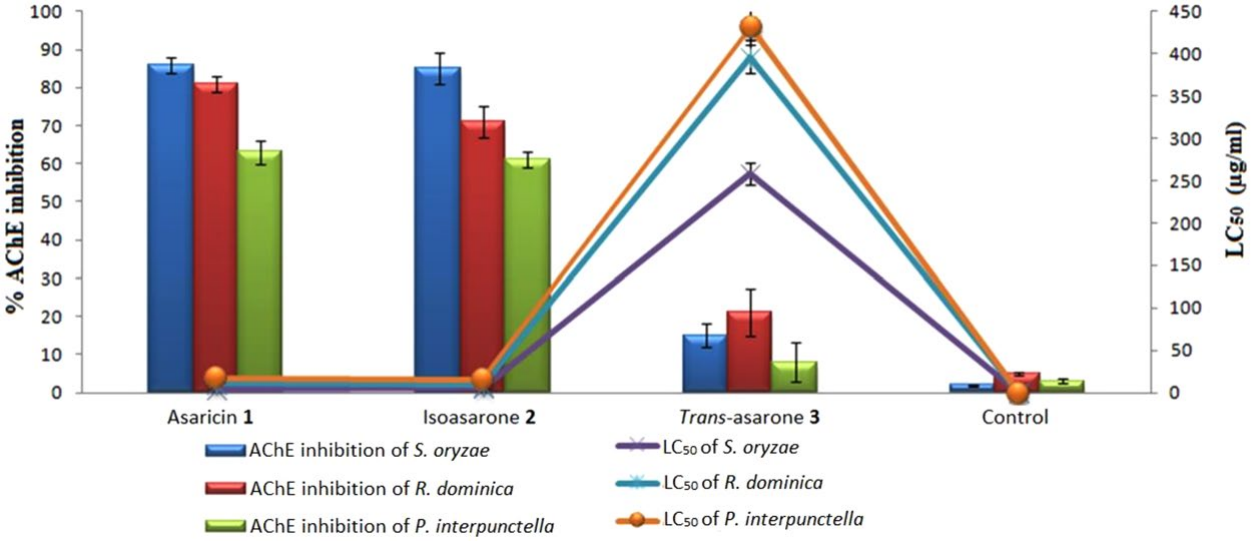
Inhibition on acetylcholinesterase enzyme by asaricin **1**, isoasarone **2** and *trans*-asarone **3** in comparison with their relative LC_50_ value on *S. oryzae*, *R. dominica* and *P. interpunctella*.

The initial AChE inhibition activity of the three compounds was evaluated at their relative LC_50_ on the enzyme supernate extract from each insect. Asaricin **1** and isoasarone **2** showed potent AChE inhibition for all three insects. Asaricin **1** and isoasarone **2** which showed high toxicity towards *S. oryzae* (LC_50_ value of 4.7 and 5.6 μg/mL respectively) also showed high AChE inhibition on enzyme supernate extracted from *S. oryzae*. Both asaricin **1** and isoasarone **2** exhibited a slightly lower AChE inhibition of the extracted enzyme from *R. dominica* and *P. interpunctella* as compared to that of *S. oryzae*. The lower AChE inhibition can relate with the relative higher LC value for *R. dominica* and *P. interpunctella*. In general, asaricin **1** exhibited the highest acetylcholinesterase inhibition followed by isoasarone **2**. *Trans*-asarone **3** exhibited a lower inhibitory activity of less than 30% as compared to asaricin **1** and isoasarone **2** for all tested insects. The LC_50_ value and percentage of AChE inhibition had significant negative correlation (Fig. 5). The correlation study between the LC_50_ and mean percentage of AChE inhibition was significant (*p* > 0.05). The results of correlation coefficient is presented in Table 6. The correlation study on AChE inhibition of *S. oryzae* was meaningful and negative for asaricin **1** and isoasarone **2** (correlation coefficient (r) of asaricin **1**: −0.920 and isoasarone **2**: −0.946) (Table 6). *R. dominica* and *P. interpunctella* correlation study was almost similar to *S. oryzae*.

**Table 6:** Correlation coefficients between AChE inhibition of *S. oryzae, R. dominica* and *P. interpunctella* and the relative LC_50_ of asaricin **1**, isoasarone **2**, and *trans*-asarone **3**.

AChE enzymes	Correlation coefficient of inhibitors		
	Asaricin **1**	Isoasarone **2**	*Trans*-asarone **3**
*S. oryzae*	−0.920*	−0.946*	−0.866*
*R. dominica*	−0.859*	−0.910*	−0.833*
*P. interpunctella*	−0.953*	−0.938*	−0.863*

*Represent the meaningful and negative correlation (*p* > 0.05).

The de-attenuated correlation coefficient was calculated using ${\rm{r}}=\frac{{\bf{n}}({\rm{\Sigma }}{\bf{xy}})-({\rm{\Sigma }}{\bf{x}})({\rm{\Sigma }}{\bf{y}})}{[{\bf{n}}{\rm{\Sigma }}{{\bf{x}}}^{2}-({\rm{\Sigma }}{{\bf{x}}}^{2})[{\bf{n}}{\rm{\Sigma }}{{\bf{y}}}^{2}-({\rm{\Sigma }}{{\bf{y}}}^{2})]}$ x represents mean percentage of AChE inhibition, y represents relative LC_95_ of asaricin **1**, isoasarone **2** and *trans*-asarone **3** against *S. oryzae, R. dominica* and *P. interpunctella*.

## Discussion

Bioassay-guided fractionation of the active hexane extract resulted in the isolation and characterization of asaricin **1**, isoasarone **2**, and *trans*-asarone **3**. Asaricin **1** and isoasarone **2** exhibited potent insecticidal activity against *S. oryzae*, *R. dominica* and *P. interpunctella*. The lowest LC_50_ and LC_95_ values for *S. oryzae* were observed for asaricin **1** and isoasarone **2** among the tested insects while *P. interpunctella* was more resistant to the toxicity of asaricin **1** and isoasarone **2**. Based on the LC_50_ values and their respective 95% confidence intervals, *trans*-asarone **3** was found to be significantly less effective towards *S. oryzae* and *R. dominica* adults and *P*. *interpunctella* larvae. The LC_50_ and LC_95_ values for all three species were variable and species specific. This result is supported by other reports, which showed great variations in the susceptibility of insects to other biopesticide product^34^. The percentage of variation in the sensitivity of stored grain insect pests is high in response to toxicity of volatile compounds. Similar results have been reported by several researches on insecticidal activities of other plant extracts^35,36^. Besides, the results of lethal time (LT_50_ and LT_95_) showed fast acting of the asaricin **1** and isoasarone **2** against the *S. oryzae*, *R. dominica* and *P. interpunctella* ^37,38^. The fast and significant mortality indicated the potent insecticidal activity of asaricin **1** and isoasarone **2**.

Furthermore, none of these compounds exhibited potent contact toxicity while asaricin **1** and isoasarone **2** were highly toxic against the pests in treated grain assay. According to previous research some natural compounds or oils express toxicity against stored product pests, such as l-carvone, d-carvone, horseradish oil, and mustard oil, but not all are active against insect pests through contact toxicity^39,40^. The results suggested that all three compounds act as stomach poison as the toxicity may be due to ingestion and digestion of the compounds in the stomach but not by absorption through insects’ curticle^41,42^. To confirm this statement, further investigations are needed after the formulation of the compounds. On the other hand, extended period of protection, repellency activities, F1 progeny activity and anti-ovipositional properties of asaricin **1** and isoasarone **2** suggested their excellent grain protectant potential.

Asaricin **1** and isoasarone **2** could provide protection against *S. oryzae*, *R. dominica* and *P. interpunctella* over a relatively long period (over 30 days) according to residual toxicity test. However, insecticidal activity of *trans*-asarone **3** showed consistent toxicity throughout the 60 days towards the three stored grain pests. This observation suggested that *trans*-asarone **3** has a longer half-life as compared to asaricin **1** and isoasarone **2**. This result may be due to stability of *trans*-asarone **3**. The three compounds have carbon double bonds in the aromatic ring and alkyl side chain. However, *trans*-asarone **3** has conjugated double bonds while asaricin **1** and isoasarone **2** have isolated double bonds. Conjugated double bond has resonance stabilization, so it is more stable than isolated double bond. Hence, *trans*-asarone **3** may be have better stability and resistance to oxidative degradation which resulted in longer half-life in comparison to asaricin **1** and isoasarone **2** ^43,44^. Although the insecticidal activity of *trans*-asarone **3** was the lowest (LC_50_ value of 258.90–432.42 μg/mL) but since its toxicity effect was consistent during the 60 days, it may be a good option to be used in mixtures with asaricin **1** and isoasarone **2** as it is most probably more stable.

**Figure 6:**
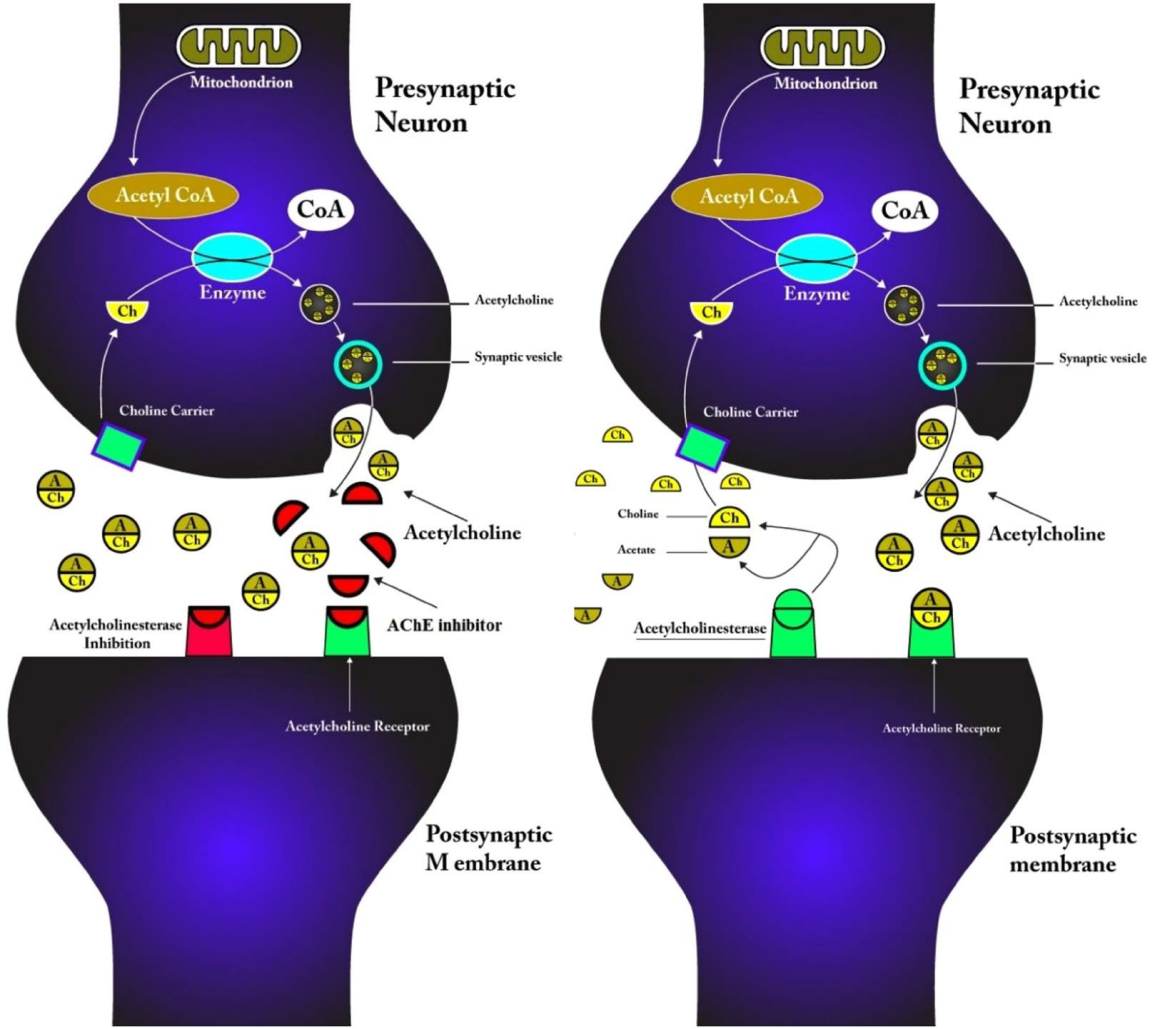
Mechanism of action of AChE with the presence of asaricin **1**, isoasarone **2** and *trans*-asarone **3** as insects acetylcholinesterase inhibitors (left) and without the presence of acetylcholinesterase inhibitors (right).

As shown in Fig. 6, the presence of asaricin **1**, isoasarone **2** and *trans*-asarone **3** as acetylcholinesterase inhibitors can cause inhibition of AChE from breaking down to neurotransmitter; acetylcholine, which disrupt or increase the duration of action of the neurotransmitter^9,11^. Mechanism of action asaricin **1** and isoasarone **2** maybe has similarity with organophosphate insecticides due to their strong AChE inhibition. The inhibition of AChE may has direct effect on bradycardia, bronchoconstriction and prolonged muscle contraction of insects which lead to paralysation and death^45^. The biochemical assay revealed the possible connection between the AChE inhibition and the toxicity of the three compounds. These results suggested that there was significant and negative relationship between AChE inhibition and LC value where higher inhibition of AChE causes stronger toxicity and results in lower LC_50_ value. The difference in the AChE inhibition for all three compounds may be due to the insect resistance and different binding modes due to differences in AChE structural features in each species studied^46,47^.

This is the first study conducted on the insecticidal activity and the mechanism of action of the compounds isolated from *P. sarmentosum* in insect pest control. The results obtained from this study suggested the potential use of *P. sarmentosum* extracts and its active components in storage pest management. In addition, further investigations regarding the safety issues for mammalian and environmental health, antifeedant activity and formulations will be conducted. Formulations may improve and enhance the insecticidal potency of the compounds.

## Materials and Methods

### General experimental procedures

General experimental procedures were similar to those described by Hematpoor *et al*.^31^.

### Plant material

The plant material was collected as previously described^31^. The voucher specimen (KU 0110) was deposited in the Herbarium of the Department of Chemistry, Faculty of Science, University of Malaya, Kuala Lumpur, Malaysia.

### Extraction and Isolation

The roots of *P. sarmentosum* were extracted as previously described^31^. The highest percentage of mortality was observed for the hexane extract in a preliminary screening of the potential toxicity of the extracts towards *S. oryzae*, *R. dominica*, and *P. interpunctella* (Table 1). Hence, the hexane extract was subjected to bioassay-guided fractionation and fraction F2 from total of eight fractions was found to exhibit the highest toxicity towards *S. oryzae*, *R. dominica*, and *P. interpunctella* (Table 1). Asaricin **1**, isoasarone **2**, and *trans*-asarone **3** were isolated from the active fraction F2 as previously described^31^.

### Structural elucidation of compounds

The structures of the isolated compounds (asaricin **1**, isoasarone **2**, and *trans*-asarone **3**) were characterized using spectroscopic data as previously described^31^ and compared with literature values^48–50^.

### Insecticidal activity

#### Test insects

All tested insects used for the experiments were obtained from colonies cultured in the laboratory at 25 ± 1 °C and 65 ± 5% relative humidity. *S. oryzae*, *R. dominica*, and *P. interpunctella* were reared on rice grains with additional 5% yeast. Two weeks old adults of *S. oryzae* and *R. dominica* and 3^rd^ instar-larvae of *P. interpunctella* were used for the bioassay.

#### Insecticidal effects on treated grain

Bioassay was conducted by dissolving 1 mg of each extract (hexane, dichloromethane and methanol) in 1 mL of acetone and introducing them onto 5 g of rice grains using a pipette. The control was only treated with 1 mL of acetone. The treated grains were dried under the fume hood. Twenty unsexed adults of *S. oryzae* and *R. dominica* were separately introduced into the respective petri dishes containing 5 g of treated rice. Twenty 3^rd^ instar larvae of *P. interpunctella* were carefully collected with a fine brush and placed in each petri dish (10 cm diameter × 1.5 cm). All treatments were monitored to obtain the mean percentage of mortality after 72 hours. Experiments were replicated four times. The same technique was used to determine the bioactivity of each fraction obtained from the active extract against the test insects.

Compounds isolated from the active fraction were evaluated for their toxicity towards *S. oryzae*, *R. dominica* and *P. interpunctella* to obtain their lethal concentration values (LC). Five grams of rice grains were treated with different concentrations (w/v) of each compound. Controls were treated with acetone. The treated grains were allowed to dry at room temperature (27 °C). The petri dishes were covered and sealed with parafilm. Mortality was recorded after 24, 48, and 72 hours of exposure.

#### Contact toxicity

Residual film bioassay technique was used to study the contact toxicity of asaricin **1**, isoasarone **2**, and *trans*-asarone **3** ^1,25^. Amount of 1 mg of each compound was dissolved in 1 mL of acetone and used as the stock solution to apply in several dosages to determine their contact toxicity. After a serial dilution, dosages between 20–200 µg/mL were carefully introduced onto filter papers (Whatman No. 1). Controls received only acetone. After drying under a fume hood for 15 minutes, each filter paper was placed at the bottom of a petri dish. Twenty adults of *S. oryzae* and *R. dominica* and twenty larvae of *P. interpunctella* were introduced into each petri dish, respectively. The petri dishes were covered and sealed with parafilm. Mortality of tested insects were recorded after 24 and 48 hours of treatment and percentage of mortality of each compound were calculated using Abbott’s formula^51^. Abbott’s formula is as follows:

1 $$(\frac{{\rm{X}}-{\rm{Y}}}{{\rm{X}}})\,\times \,100$$

where:

X = percent live in control.

Y = percent live in treatment.

#### Residual toxicity against storage pests

Residual toxicity against adults of *S. oryzae* and *R. dominica*, and larvae of *P. interpunctella* were assisted by optimization of the method from Islam and Talukder, 2005^52^. The assay was carried out in separate plastic cups for a period of 60 days after 15 g of rice were treated with asaricin **1**, isoasarone **2**, and *trans*-asarone **3**. The length of exposure and the dosage of each compound were chosen as the concentration required to cause 95% mortality of the individuals. The LC_95_ values obtained from the earlier study (section 2.5.2) was used to evaluate the persistence of toxicity during the 60 days period. After each treatment was completed, the plastic cups were closed with their caps, secured and held at room temperature (27 °C). The control was only treated with acetone. These bioassays were carried out over a period of 60 days whereby on the 7^th^ day, 30 insects were placed in each plastic cup. After 72 hours, the insects were removed from each cup. Insect’s mortality was recorded and the dead insects were discarded. All cups were tightly closed until the next bioassay. The procedures were repeated on the 15^th^, 20^th^, 30^th^, 40^th^, 50^th^ and 60^th^ day. All bioassays were replicated 4 times.

#### Repellency test

Repellency of asaricin **1**, isoasarone **2**, and *trans*-asarone **3** was evaluated using area preference methods^25^. Whatman No. 1 filter papers were cut into halves. One side treated with 100 µL/mL of asaricin **1**, isoasarone **2** and *trans*-asarone **3** respectively using pipette. The other half was treated with acetone as control. The treated and control half discs were air dried under laboratory conditions and full discs re-made using paper tapes to attach together. Each paper was placed in a petri dish and ten *S. oryzae* was introduced at the centre of the filter paper then the petri dish was closed and sealed with parafilm. Each treatment was replicated four times and the number of *S. oryzae* present on each side was recorded every 5 hours up to 20 hours. The same procedures were applied for *R. dominica* and *P. interpunctella*. The Percentage of Repellency (PR) calculated as follows:

2 $${\rm{PR}}=\frac{{N}_{c}-{N}_{t}}{{N}_{c}+{N}_{t}}\times 100\, \% $$

where:

PR = Percentage of repellency.

N_c_ = Number of insects present on the control strip.

N_t_ = Number of insects present on the treated strip.

Negative PR value were treated as zero.

#### *F1 progeny activity of* asaricin 1, isoasarone 2, and *trans*-asarone 3

Fifty grams of rice grains were weighed and then three concentrations (1, 2, and 4 μg/mL) of each compound asaricin **1**, isoasarone **2**, and *trans*-asarone **3** were individually mixed and specimen were deposited in a 300 mL of plastic containers. The compounds were dissolved in acetone and uniformly spread on rice. Rice treated with acetone only was set as control. Ten pairs of adult insects were introduced into each container. Four replicates of each treatment and untreated controls were laid out in Complete Randomized Design. Adult mortality was counted and recorded after 48 and 72 hours of application. On the 4^th^ day, all insects whether dead or alive, were removed and experiments were left for 35 days to allow for emergence of F1 generation. The number of adults emerged was counted after 35 days and inhibition rate (% IR) in adult emergence was calculated using following formula^53^.

3 $$ \% \,{\rm{IR}}=\frac{{C}_{n}-{T}_{n}}{{C}_{n}}\times 100\, \% $$

where:

C_n_ = number of emerged insects in the control.

T_n_ = number of emerged insects in the treated container.

#### Oviposition deterrent activity

The asaricin **1**, isoasarone **2**, and *trans*-asarone **3** were tested for their oviposition inhibition activity^54^. Three concentrations (0.5, 1, 2, 3, and 4 μg/mL) for each compound were prepared by dissolving them in acetone. Twenty undamaged grain of rice were placed in the plastic petri dish and treated separately with different dose of asaricin **1**, isoasarone **2**, and *trans*-asarone **3** in uniform manner. The seeds were air dried until all acetone evaporated. For control seeds were dressed in 1 mL acetone only. The treated samples were kept in laboratory condition (27 ± 2 °C and 80 ± 5% RH). Five males and five females were placed on each treatment. After 48 hours the mortality of each insect was observed in each petri dish and all insects (live and dead) were removed. The number of eggs laid on seeds of treated and control seeds were counted after three days of starting the experiment. To evaluate the number of eggs laid for *S. oryzae* on rice grains samples were recorded after staining the grains to detect the egg plugs^55^.

4 $$ \% \,{\rm{OD}}=\frac{{C}_{s}-{T}_{s}}{{C}_{s}}\times 100\, \% $$

where:

C_s_ = number of eggs laid on the control.

T_s_ = number of eggs laid in the treated container.

### Biochemical assays

The tested insects were respectively subjected to acetylcholinesterase biochemical assay (AChE)^56^. Each insect was removed and homogenized at 0 °C in 0.05 M-phosphate buffer, pH 7.5 (KH_2_PO_4_-NaOH) (Sigma) using a glass homogenizer pestle (5%, w/v, homogenate). Plastic tube covered with ice were used to keep the homogenized mixture. The homogenates were centrifuged at 16,000 rpm for 20 min (5 °C). These supernatants were kept in the ice and used without further purification for studying the acetylcholinesterase and its inhibition. To determine the AChE inhibition activity, procedures from Ellman *et al*., 1961 were slightly modified and used^57,58^. 10 μL of enzyme supernatants were transferred to each well of a 96-well microtiter plate using electronic multichannel pipette (Eppendorf, USA) then mixed with 20 μL of tested compounds in solution form and 150 μL of phosphate buffer which were kept at 1–4 °C temperature were added. Asaricin **1**, isoasarone **2**, and *trans*-asarone **3** were dissolved in DMSO (Merck, USA) as AChE inhibitor at their LC_95_ value and 0.1% DMSO (v/v) were added into test well in separated rows. The microtiter plates were incubated for 10 minutes at 25 °C. Next step was followed by adding 20 μL of acetylthiocholine iodide (ACTHI) (Sigma-Aldrich, USA) (0.4 mM) and DTNB (Sigma-Aldrich, USA) (0.3 mM) to enzyme supernatants solution to observe the reaction. During the 30 minutes reaction at room temperature, yellowish or colorless solution was observed. The microtiter plates were sentenced for microplate readers (Synergy H1 Hybrid Multi-Mode Microplate Reader, USA) at 412 nm and results were recorded. Percentage of inhibition calculated based on the mean optical density of enzyme as below:

5 $${\rm{ \% }}\,{\rm{I}}{\rm{n}}{\rm{h}}{\rm{i}}{\rm{b}}{\rm{i}}{\rm{t}}{\rm{i}}{\rm{o}}{\rm{n}}=[1-(\frac{{\rm{A}}{\rm{b}}{\rm{s}}{\rm{o}}{\rm{r}}{\rm{b}}{\rm{a}}{\rm{n}}{\rm{c}}{\rm{e}}\,{\rm{s}}{\rm{a}}{\rm{m}}{\rm{p}}{\rm{l}}{\rm{e}}-{\rm{A}}{\rm{b}}{\rm{s}}{\rm{o}}{\rm{r}}{\rm{b}}{\rm{a}}{\rm{n}}{\rm{c}}{\rm{e}}\,{\rm{o}}{\rm{f}}\,{\rm{b}}{\rm{a}}{\rm{c}}{\rm{k}}{\rm{g}}{\rm{r}}{\rm{o}}{\rm{u}}{\rm{n}}{\rm{d}}}{{\rm{A}}{\rm{b}}{\rm{s}}{\rm{o}}{\rm{r}}{\rm{b}}{\rm{a}}{\rm{n}}{\rm{c}}{\rm{e}}\,{\rm{o}}{\rm{f}}\,\,{\rm{b}}{\rm{l}}{\rm{a}}{\rm{n}}{\rm{k}}-{\rm{A}}{\rm{b}}{\rm{s}}{\rm{o}}{\rm{r}}{\rm{b}}{\rm{a}}{\rm{n}}{\rm{c}}{\rm{e}}\,{\rm{o}}{\rm{f}}\,{\rm{b}}{\rm{a}}{\rm{c}}{\rm{k}}{\rm{g}}{\rm{r}}{\rm{o}}{\rm{u}}{\rm{n}}{\rm{d}}\,})]\,\times \,100{\rm{ \% }}$$

The resulting data for each selected insect was analyzed using correlation study compare the enzyme expression levels between different insects in response to asaricin **1**, isoasarone **2**, and *trans*-asarone **3**. All levels of statistical significance were determined at *p* < 0.05.

### Data Analysis

Bioassay data within the range of 5–95% were pooled and subjected to probit analysis^59^ by using Polo Plus (LeOra Software, Berkeley, CA) to obtain 50% and 95% lethal concentrations (LC_50_ and LC_95_). Data on adult mortality were also subjected for determining lethal time required by each isolated compound to kill 50% and 95% (LT_50_ and LT_95_). Significant differences in the LC_50_ and LC_95_ values were based on non-overlap of 95% confidence intervals^60^. Abbott’s formula^51^ was applied to correct the percentage of mortality if the control mortality was more than 5%. The analyses of the data were done using ANOVA followed by Tukey’s test using SAS version 9.1.3, 2002 software. Statistical correlation study was conducted to understand the relation between acetylcholinesterase activity and LC_50_ dose of asaricin **1**, isoasarone **2**, and *trans*-asarone **3** levels of statistical significant were determined at *p* < 0.05.

### Data availability statement

All data generated or analysed during this study are included in this published article.

## References

[1] Rajashekar, Y. & Shivanandappa, T. A novel natural insecticide molecule for grain protection. *Julius-Kühn-Archiv*, 910–915, 10.5073/jka.2010.425.413 (2010).

[2] Rojht H, Košir IJ, Trdan S (2012). Chemical analysis of three herbal extracts and observation of their activity against adults of *Acanthoscelides obtectus* and *Leptinotarsa decemlineata* using a video tracking system. J. Plant Dis. Protect..

[3] Nakakita, H. *Stored rice and stored product insects*. (A.C.E. Corporation, Tokyo, 1998).

[4] Majeed MZ (2015). Biology and management of stored products’ insect pest *Rhyzopertha dominica* (Fab.) (Coleoptera: Bostrichidae). Int J. Biosci..

[5] Chanbang Y, Arthur FH, Wilde GE, Throne JE, Subramanyam B (2008). Susceptibility of eggs and adult fecundity of the lesser grain borer, *Rhyzopertha dominica*, exposed to methoprene. J. Insect Sci..

[6] Nellie, W. S. C. *Development And Competition In Rice Weevil, Sitophilus Oryzae (L.) And Red Flour Beetle, Tribolium Castaneum (Herbst), And Their Responses To Active Compounds In Selected Spices* Doctor of Philosophy thesis, Universiti Sains Malaysia, (2016).

[7] Fasulo, T. R. & Knox, M. A. (University of Florida, University of Florida, 1998).

[8] Mohandass S, Arthur FH, Zhu KY, Throne JE (2007). Biology and management of *Plodia interpunctella* (Lepidoptera: Pyralidae) in stored products. J. Stored Prod. Res..

[9] Čolović MB, Krstić DZ, Lazarević-Pašti TD, Bondžić AM, Vasić VM (2013). Acetylcholinesterase Inhibitors: Pharmacology and Toxicology. Curr. Neuropharmacol..

[10] Rajashekar Y, Raghavendra A, Bakthavatsalam N (2014). Acetylcholinesterase Inhibition by Biofumigant (Coumaran) from Leaves of *Lantana camar*a in Stored Grain and Household InsectPests. BioMed Res. Int..

[11] Fukuto TR (1990). Mechanism of action of organophosphorus and carbamate insecticides. Environ. Health Perspect..

[12] Nabeshima T (2004). An amino acid substitution attributable to insecticide-insensitivity of acetylcholinesterase in a Japanese encephalitis vector mosquito. Culex tritaeniorhynchus. Biochem. Biophys. Res. Commun..

[13] Cheng S-S, Chang H-T, Chang S-T, Tsai K-H, Chen W-J (2003). Bioactivity of selected plant essential oils against the yellow fever mosquito *Aedes aegypti* larvae. Bioresour. Technol..

[14] Moye JK, Pritsos CA (2010). Effects of Chlorpyrifos and Aldicarb on Flight Activity and Related Cholinesterase Inhibition in Homing Pigeons, Columba livia: Potential for Migration Effects. Bull. Environ. Contam. Toxicol..

[15] Arthur FH (1996). Grain protectants: Current status and prospects for the future. J. Stored Prod. Res..

[16] Adakole JA, Adeyemi AFF (2012). Larvicidal effects of cymbopogon citratus (lemon grass) extract against *Culex quinquefasciatus* qularvae (Diptera, culicidae). IJAES.

[17] Golob, P. & Gudrups, I. The use of spices and medicinals as bioactive protectants for grains. (Food and Agriculture Organization of the United Nations Rome, FAO Agricultural Sciences Bulletin No. 137, 1999).

[18] Lale, N. E. S. An overview of the use of plant products in the management of stored product Coleoptera in the tropics. *Postharvest News and Information* **6** (1995).

[19] Koul O, Walia S, Dhaliwal GS (2008). Essential Oils as GreenPesticides: Potential and Constraints. Biopestic. Int..

[20] Tavares WS (2010). Selective effects of natural and synthetic insecticides on mortality of *Spodoptera frugiperda* (Lepidoptera: Noctuidae) and its predator *Eriopis connexa* (Coleoptera: Coccinellidae). J. Environ. Sci. Health. B.

[21] Grdiša M, Gršić K (2013). Botanical Insecticides in Plant Protection. Agric. Conspec. Sci..

[22] Belmain SR, Golob P, Andan HF, Cobbinah JR (1999). Ethnobotanicals—future prospects as post-harvest insecticides. Agro Food Ind. Hi Tech.

[23] Ahmed, S. & Koppel, B. In *Proceedings of the American Association for the Advancement of Science Annual Meeting*.

[24] Rajashekar Y, Bakthavatsalam N, Shivanandappa T (2012). Botanicals as Grain Protectants. Psyche.

[25] Obeng-Ofori D, Reichmuth CH, Bekele AJ, Hassanali A (1998). Toxicity and protectant potential of camphor, a major component of essential oil of *Ocimum kilimandscharicum*, against four stored product beetles. Int. J. Pest Manag..

[26] Damsud T, Adisakwattana S, Phuwapraisirisan P (2013). Three new phenylpropanoyl amides from the leaves of *Piper sarmentosum* and their α-glucosidase inhibitory activities. Phytochem. Lett..

[27] Chan EWC, Wong SK (2014). Phytochemistry and pharmacology of thre*e Pipe*r species. An update..

[28] Zainal Ariffin SH (2009). Intrinsic anticarcinogenic effects of *Piper sarmentosum* ethanolic extract on a human hepatoma cell line. Cancer Cell Int..

[29] Zoubiri S, Baaliouamer A (2014). Potentiality of plants as source of insecticide principles. J. Saudi Chem. Soc..

[30] Chansang U (2005). Mosquito larvicidal activity of aqueous extracts of long pepper (*Piper retrofractum* vahl) from Thailand. J Vector Ecol..

[31] Hematpoor A (2016). Inhibition and Larvicidal Activity of Phenylpropanoids from *Piper sarmentosum* on Acetylcholinesterase against Mosquito Vectors and Their Binding Mode of Interaction. PLoS ONE.

[32] Intirach J (2012). Chemical Constituents and Combined Larvicidal Effects of Selected Essential Oils against *Anopheles cracens* (Diptera: Culicidae). Psyche.

[33] Chaithong U (2006). Larvicidal effect of pepper plants on *Aedes aegypti* (L.) (Diptera: Culicidae). Journal of Vector Ecology.

[34] Shaaya E, Kostjukovski M, Eilberg J, Sukprakarn C (1997). Plant oils as fumigants and contact insecticides for the control of stored-product insects. J. Stored Prod. Res..

[35] Koul O (2004). Biological activity of volatile di-n-propyl disulfide from seeds of neem, *Azadirachta indica* (Meliaceae), to two species of stored grain pests, *Sitophilus oryzae* (L.) and *Tribolium castaneum* (Herbst). J. Econ. Entomol..

[36] Huang Y, Tan JMWL, Kini RM, Ho SH (1997). Toxic and antifeedant action of nutmeg oil against *Tribolium castaneum* (Herbst) and *Sitophilus zeamais* Motsch. J. Stored Prod. Res..

[37] Muhamad R, Dzolkhifli O (1995). Evaluation of three bioassays for detecting resistance in cocoa mirid, *Helopeltis theivora*. Resistant Pest Management Newsletter.

[38] Busvine, J. R. *Recommended methods for measurement of pest resistance to pesticides*. (Food and Agriculture Organization of the United Nations, 1980).

[39] Kim S-I, Park C, Ohh M-H, Cho H-C, Ahn Y-J (2003). Contact and fumigant activities of aromatic plant extracts and essential oils against *Lasioderma serricorne* (Coleoptera: Anobiidae). J. Stored Prod. Res..

[40] Tripathi AK, Prajapati V, Kumar S (2003). Bioactivities of l-carvone, d-carvone, and dihydrocarvone toward three stored product beetles. J. Econ. Entomol..

[41] Shukla E, Thorat LJ, Nath BB, Gaikwad SM (2015). Insect trehalase: Physiological significance and potential applications. Glycobiology.

[42] Asano N (2003). Glycosidase inhibitors: update and perspectives on practical use. Glycobiology.

[43] Ouellette, R. J. & Rawn, J. D. *Organic Chemistry: Structure, Mechanism, and Synthesis*. (Elsevier Science, 2014).

[44] Bhardwaj A (2010). Larvicidal and Structure–Activity Studies of Natural Phenylpropanoids and Their Semisynthetic Derivatives against the Tobacco Armyworm Spodoptera litura (Fab.) (Lepidoptera: Noctuidae). Chem. Biodivers..

[45] Nair VP, Hunter JM (2004). Anticholinesterases and anticholinergic drugs. Contin. Educ. Anaesth. Crit. Care Pain.

[46] Massoulie J, Bon S (1982). The Molecular Forms of Cholinesterase and Acetylcholinesterase in Vertebrates. Ann. Rev. Neurosci..

[47] Arpagaus M, Fournier D, Toutant J-P (1988). Analysis of acetylcholinesterase molecular forms during the development of *Drosophila melanogaster*. Evidence for the existence of an amphiphilic monomer. Insect Biochem..

[48] Tanimori S, Watanabe K, Kirihata M (2009). Synthesis of cinnamyl-sesamol derivatives. Res. Chem. Intermed..

[49] Santos BVdO, Chaves EVLd-CMCdO, Gray AI (1998). Phenylalkanoids from piper Marginatumfn2. Phytochemistry.

[50] Patra A, Mitra AK (1981). Constituents of *Acorus calamus*: Structure of Acoramone. Carbon-13 NMR Spectra of *Cis*- and *Trans*-Asarone. J. Nat. Prod..

[51] Abbott WS (1925). A Method of Computing the Effectiveness of an Insecticide. J. Econ. Entomol..

[52] Islam MS, Talukder FA (2005). Toxic and residual effects of *Azadirachta indica*, *Tagetes erecta* and *Cynodon dactylon* seed extracts and leaf powders towards Tribolium castaneum. JPDP.

[53] Tapondjou LA, Adler C, Bouda H, Fontem DA (2002). Efficacy of powder and essential oil from *Chenopodium ambrosioides* leaves as post-harvest grain protectants against six-stored product beetles. J. Stored Prod. Res..

[54] Chudasama JA, Sagarka NB, Sharma S (2015). Deterrent effect of plant extracts against *Callosobruchus maculatus* on stored cowpea in Saurashtra (Gujarat, India). IJANS.

[55] Holloway GJ (1986). The potency and effect of phytotoxins within yellow split-pea (*Pisum sativum*) and adzuki bean (*Vigna angularis*) on survival and reproductive potential of *Sitophilus oryzae* (L.) (Coleoptera: Curculionidae). Bulletin of Entomological Research.

[56] WHO. Techniques to detect insecticide resistance mechanisms (field and laboratory manual) 42 (World Health Organization, Geneva, Switzerland, 1998).

[57] Ellman GL, Courtney KD, Andres V, Featherstone RM (1961). A new and rapid colorimetric determination of acetylcholinesterase activity. Biochem. Pharmacol..

[58] Swale, D. R. *et al*. Inhibitor profile of bis(n)-tacrines and N-methylcarbamates on acetylcholinesterase from *Rhipicephalus* (Boophilus) *microplus* and *Phlebotomus papatasi*. *Pest Biochem. Physiol*. **106**, 10.1016/j.pestbp.2013.1003.1005, 10.1016/j.pestbp.2013.03.005 (2013). 10.1016/j.pestbp.2013.03.005 PMC3811934 24187393

[59] Finney, D. J. *Probit analysis*. (Cambridge, England: Cambridge University Press, 1971).

[60] Kljajić P, Perić I (2007). Effectiveness of wheat-applied contact insecticides against *Sitophilus granarius* (L.) originating from different populations. J. Stored Prod. Res..

